# Layer Thickness Effects on Residual Stress, Microstructure, and Tensile Properties of Cu18150/Al1060/Cu18150 Multilayered Composites: An Integrated EBSD-KAM Approach

**DOI:** 10.3390/ma18204673

**Published:** 2025-10-11

**Authors:** Yuchao Zhao, Mahmoud Ebrahimi, Shokouh Attarilar, Qiang Lu, Haiyan Jiang, Qudong Wang

**Affiliations:** 1 National Engineering Research Center of Light Alloy Net Forming and Key State Laboratory of Metal Matrix Composites, School of Material Science and Engineering, Shanghai Jiao Tong University, Shanghai 200240, China; zhaoyuchao@sjtu.edu.cn (Y.Z.); ebrahimi@maragheh.ac.ir (M.E.); sh.attarilar@yahoo.com (S.A.); luqiang951127@sjtu.edu.cn (Q.L.); jianghy@sjtu.edu.cn (H.J.); 2 Department of Mechanical Engineering, Faculty of Engineering, University of Maragheh, Maragheh 83111-55181, Iran; 3 Department of Materials Engineering, Faculty of Engineering, University of Maragheh, Maragheh 83111-55181, Iran

**Keywords:** Intermetallics, grain refinement, EBSD-KAM approach, layer thickness, microstructure evolution, mechanical properties

## Abstract

This study examines the influence of layer thickness (0.9, 1.6, 2.4, and 4 mm) on the distribution of residual stress, microstructural evolution, and tensile properties of Cu18150/Al1060/Cu18150 multilayered composites fabricated via a combined cast-rolling and hot-rolling technique. The grain refinement, dislocation density, and residual stress gradients across the interfaces were characterized and analyzed using integrated electron backscatter diffraction and kernel average misorientation mapping. The results demonstrated that specimens with a lower layer thickness (0.9–1.6 mm) possess a significantly improved tensile strength of 351 MPa, which is mainly due to the significant grain refinement and the presence of compressive residual stresses at the region of the Al/Cu interfaces. However, tensile strength decreased to 261 MPa in specimens with thicker layers (4 mm), accompanied by improved ductility, e.g., elongation of 30%. This is associated with a reduction in the degrees of interfacial constraint and the formation of more homogeneous deformation structures that accommodate a larger strain. The intermediate layer thickness of 2.4 mm offers an optimal compromise, achieving a tensile strength of 317 MPa while maintaining balanced mechanical performance. These results emphasize the importance of layer thickness in controlling such stress profiles and optimizing the mechanical behavior of hybrid metal composites, providing useful guidance on the design and fabrication of superior structural-form materials.

## 1. Introduction

Multilayered metallic composites (MMCs) that include different materials such as copper and aluminum are potentially attractive for lightweight structure applications as a result of the synergistic effects of high strength, promising electrical conductivity, and improved corrosion resistance [1,2]. Multilayered metallic composites, such as Cu/Al/Cu, have attracted considerable interest for engineering applications because they can integrate desirable properties of different metals while compensating for their drawbacks. Strength can be enhanced and ductility preserved by the stratified architecture (where the interfaces serve as barriers for dislocation movement) [3,4]. The interfacial interactions among layers can also resist crack growth and improve toughness. In Cu/Al/Cu composites, copper offers high electrical conductivity; also, the outer copper layer may offer better corrosion resistance in some applications than that of naked aluminum [5,6,7]. The low density and cost of aluminum match well with copper’s excellent electrical and thermal conductivity. By applying two layers of Cu and Al together, engineers consider both performance and low cost [8,9,10]. Overall, Cu/Al/Cu metallic composites exemplify how layered material design can address engineering challenges, offering tailored solutions that single-phase materials cannot achieve. Huang et al. [11] demonstrated that interface density in Cu/Nb nanolaminates directly modulates dislocation storage capacity, with thinner layers achieving 2–3 times higher yield strength than monolithic counterparts. Zheng et al. [12] reported similar Hall-Petch strengthening in Al/Cu composites, where interface spacing below 50 µm amplified grain boundary hardening [9,13]. Recent studies have provided valuable insights into the microstructural control and mechanical behavior of Cu-Al multilayer composites and bimetal systems. Their investigations emphasize the critical role of interfacial intermetallic phases such as Al_4_Cu_9_ and Al_2_Cu, demonstrating that managing these phases through controlled processing can enhance strength and toughness by tailoring phase thickness below critical thresholds. For example, limiting intermetallic thicknesses to under 1 μm allowed improvements in tensile strength of up to 15%, highlighting the importance of precise interface engineering [14,15,16,17]. Furthermore, they have shown how diffusion bonding parameters, including temperature and holding time, influence grain boundary characteristics and residual stress distributions within Cu-Al composites, which in turn strongly affect ductility by altering strain accommodation mechanisms. These findings align with observations in our study, where layer thickness-dependent microstructural evolution—including grain refinement and residual stress profiles—modulates the strength-ductility balance in Cu18150/Al1060/Cu18150 multilayers. Integrating these results highlights the potential for interface design strategies to tailor multilayered composites for targeted mechanical performance [16,17,18,19].

The mechanical properties and microstructural characterization of multilayered metal laminates and composites (e.g., Cu/Al, Al/Ni, Ti/Al) are strongly influenced by individual layer thickness, which affects dislocation motion, interfacial behavior, and overall strength-ductility balance [13,20,21,22]. Several studies have investigated these effects using electron backscatter diffraction (EBSD) and tensile testing, providing key insights into deformation mechanisms. In titanium-carbon laminates, varying titanium layer thickness (0.3–0.5 mm) at constant total thickness (2.5 mm) showed minimal impact on low-velocity impact resistance. Thicker titanium layers (0.5 mm), however, decreased force fluctuations during impact, implying more consistent energy absorption [10,23,24]. Furthermore, studies on Al/5wt.%Fe_2_O_3_ composites made by selective laser melting (SLM) revealed that the microstructure is much influenced by layer thickness. Enhanced oxygen removal from thicker layers (75 µm) produced an aluminum matrix saturated with iron and stronger intermetallic phases (e.g., Al-Fe oxides). Compared to thinner layers (50 µm), these phases help to produce more microhardness and solution hardening [20,25,26]. Reducing layer thickness enhanced energy absorption during impact testing, according to studies on AA6061/AA7075 laminated composites. Proper stress redistribution across interfaces is made possible by thinner layers, leading to improved fracture toughness [27,28]. Also, adjusting layer thickness ratios in TiB/Ti-based ceramic-metal composites changed stress redistribution under bending loads. Thinner ceramic layers provided higher elastic recovery, while thicker metallic layers improved load-bearing capacity [29,30,31].

In the matter of metal composites, residual stresses arising from differential thermal expansion and plastic deformation during layer stacking remain a critical challenge, often compromising interfacial integrity and mechanical performance [32,33]. In the study conducted by Li et al. [34], compressive stresses in cold-rolled Cu/Al clads were linked to thermal expansion mismatch and plastic deformation incompatibility. Recent studies on polymer-mineral [1] and ceramic-metal [32] multilayers highlight that layer thickness governs stress gradients and damage tolerance; yet, systematic investigations on metallic systems—particularly Cu/Al/Cu—are scarce. Current approaches frequently focus on isolated aspects (for example, microstructure or mechanical testing) rather than integrating techniques such as EBSD, X-ray diffraction (XRD), and tensile testing to comprehensively understand layer thickness effects. High-precision mapping of localized plastic strain and grain misorientation, which are made possible by advances in EBSD-KAM analysis, offers insights into deformation mechanisms inaccessible with traditional microscopy. Kernel average misorientation (KAM), derived from EBSD data, is increasingly used to assess residual stress by quantifying local lattice distortions and plastic strain gradients. However, systematic investigations into the application of KAM for analyzing residual stresses in multilayer metallic composites have remained limited. The mapping of local misorientations within grains, which correspond to plastic deformation and residual stress, is accomplished using KAM values.

Higher dislocation density and localized stress concentrations are generally indicated by higher KAM values. This method works effectively for multilayer systems where mismatched mechanical or thermal expansion causes stress gradients at interfaces. While ANSYS (version 2023 R2) modeling of Si_3_N_4_/SiC composites [32] quantifies stress-driven fracture modes, KAM-based studies on nickel alloys [35] and cemented carbides show correlations between misorientation gradients and residual stress magnitudes. Still, relatively little is known about how layer thickness, residual stress, and tensile characteristics interact in roll-bonded MMCs. A study examined the evolution of residual stress during thermal cycling between 25 and 400 °C using Cu/Nb nanoscale metallic multilayers adhered to Si substrates [36,37,38]. The study emphasized how interfacial density and layer thickness affect thermoelastic stress responses. Curvature analysis and X-ray diffraction peak shifts were used to measure stress, and the results indicated that Nb layers were more influential in the development of stress than Cu layers. The results highlighted the significance of interfacial density in stress evolution by demonstrating that nanoscale multilayers display stress behaviors comparable to those of their monolithic counterparts [39,40]. Composite systems with metallic inclusions or multilayer architectures can also be subjected to residual stress analysis. Research has used data derived from EBSD to map stress distributions affected by thermal cycling or fabrication processes. These methods are useful for discovering regions where high concentrations of residual stress can be expected to cause failure [41,42]. By investigating Cu18150/Al1060/Cu18150 composites with different layer thicknesses, the presented work fills in the aforementioned knowledge gaps. The primary objective of this study is to systematically investigate how varying layer thicknesses (0.9, 1.6, 2.4, and 4.0 mm) influence the microstructural evolution, residual stress distribution, and tensile mechanical properties of Cu18150/Al1060/Cu18150 multilayered composites fabricated via combined cast-rolling and hot-rolling techniques. By integrating advanced microstructural characterization methods such as EBSD and kernel average misorientation mapping with mechanical testing, this work aims to elucidate the interplay between processing, layer architecture, and performance. The insights gained will provide fundamental guidance for optimizing multilayer metal composites with enhanced strength-ductility balance and structural integrity.

## 2. Materials and Methods

### 2.1. Material Fabrication

The Cu18150/Al1060/Cu18150 multilayered composites were fabricated using a combined cast-rolling and hot-rolling technique designed to achieve strong metallurgical bonding and desired layer thicknesses. The bonding between Cu and Al layers was accomplished primarily through diffusion bonding activated by controlled thermal and mechanical conditions. Interfaces were carefully prepared by surface cleaning and roughening prior to bonding to promote effective atomic contact and reduce oxide contamination. The rolling procedures involved multiple stages to progressively reduce thickness and refine the microstructure. Initial cast rolling was performed at a temperature of approximately 690 °C for Cu and 500 °C for Al, followed by hot rolling at around 250 °C using an industrial four-high rolling mill. A carefully controlled symmetric rolling was conducted under the following parameters, including a per-pass reduction of approximately 35%, rolling speed maintained near 5.0 m/min, and precise roll gap control (±10 µm). A hydraulic roll bending system was engaged to compensate for roll crown and ensure uniform deformation across the sheet width. These rolling and bonding conditions were optimized to enhance interface quality, minimize the formation of brittle intermetallic phases, and achieve homogeneous deformation without delamination or cracking. The layered architecture with tailored thicknesses of 0.9, 1.6, 2.4, and 4.0 mm was attained through this precise processing sequence, which was monitored using embedded thermocouples and surface quality inspections. The multilayered composites were fabricated using an industrial four-high rolling mill equipped with 350 mm diameter tungsten carbide work rolls (surface finish 0.2 µm) and 940 mm diameter forged steel backup rolls with 65 HRC hardness, sourced from Shanghai Orient Metallurgical Equipment Ltd., China. The rolling mill also featured a hydraulic roll bending system for crown compensation and employed a closed-loop servo system that precisely controlled the rolling speed at 2.0 m/min ±0.05. The chemical composition of the 1060 aluminum and 18150 copper alloys (provided by Shanghai Miandi Metal Group Co., Ltd., Shanghai, China) was measured via spark direct-reading spectrometry and is detailed in Table 1, as used in [43]. The choice of Cu-Cr-Zr alloy over pure copper was driven by its superior strength and conductivity. Cu-Cr-Zr alloys are extensively used in various sectors, such as aerospace applications, high-speed railway electrical contact wires, and integrated circuit lead frames.

To obtain the required final thickness variations, the composite material with a thickness of approximately 5.4 mm underwent a secondary cold roll-bonding process. Using a two-high rolling mill with delicately regulated parameters, this cold-working step was carried out at room temperature (25 °C). Multiple passes were carried out within the rolling sequence, and each incremental reduction was carefully kept at around 30% of the current thickness to guarantee uniform deformation and avoid edge cracking or delamination. This method of progressive reduction allowed the material to be gradually reduced from its original thickness to the desired final thicknesses of 0.9, 1.6, 2.4, and 4 mm.

Two crucial purposes were fulfilled by the cold roll-bonding process: first, it allowed for exact control over the final layer thicknesses needed for the experimental matrix; second, it strengthened the interfacial bonding between the Cu18150 and Al1060 layers by severe plastic deformation of the bonding interfaces. The material was routinely examined for dimensional consistency and surface quality in between rolling passes. The composite material was subjected to an intermediate annealing treatment at 400 °C for two hours in an argon atmosphere after the last cold-rolling pass. These thermal processing steps were used to (i) relieve residual stresses that had accumulated during cold working; (ii) promote interfacial diffusion bonding without excessive intermetallic formation; and (iii) partially recrystallize the severely deformed microstructure while preserving the intended layer thicknesses.

A strategic roll-bonding technique was used to reduce the copper alloy thickness ratio inside the composite structure as intended. A symmetrical trilayered composite architecture (Cu18150/Al1060/Cu18150) was developed during the fabrication process by hot roll-bonding an extra Cu18150 layer with the pre-existing Cu18150/Al1060 bimetal. The optimization of the copper-to-aluminum thickness ratio while preserving structural integrity was the specific goal of this methodology.

Both material components underwent careful surface preparation before the roll-bonding operation: (i) degreasing procedure: the produced Cu18150/Al8011 bimetallic sheets and extra Cu18150 plates were thoroughly cleaned with a solvent that included immersion in analytical-grade acetone for 10 min while being agitated by ultrasonic waves at a frequency of 40 kHz. To eliminate any remaining impurities, they were then rinsed in ethanol; and (ii) the process of surface roughening involves mechanical abrasion with stainless steel wire brushes (0.3 mm diameter bristles) and controlled brushing parameters: a rotational speed of 2000 rpm and a contact force of 5 N. White light interferometry (Wyko NT1100, Micro-Epsilon, Offenburg, Germany) is used to evaluate surface roughness, and the final surface roughness is maintained at Ra < 2 μm (average of 5 measurements). It should be noted that the dimensional specifications of the starting materials were precisely controlled: Cu18150/Al8011 bimetallic sheets of 400 (L) × 200 (W) × 3.9 (T) mm and additional Cu18150 plates of 400 (L) × 200 (W) × 1.5 (T) mm.

To accomplish metallurgical bonding between the component layers, the prepared sheet assembly was then subjected to a carefully regulated hot roll-bonding procedure. In order to guarantee ideal interface properties while preserving material integrity, the rolling parameters were methodically adjusted through early trials. At three points along the length of the sheet, embedded K-type thermocouples were used to monitor the rolling process, which was carried out at a precisely controlled temperature of 250 °C (±5). This temperature was selected based on

- Being below the solidus temperatures of both Cu18150 (1083 °C) and Al1060 (660 °C) to prevent partial melting.
- Providing sufficient thermal activation for diffusion bonding (Q ≈ 0.4–0.6 Tm),
- Minimizing oxide formation (oxidation rates increase exponentially above 300 °C),
- Optimizing intermetallic compound (IMC) formation at the interface [44,45].

**Figure 1:**
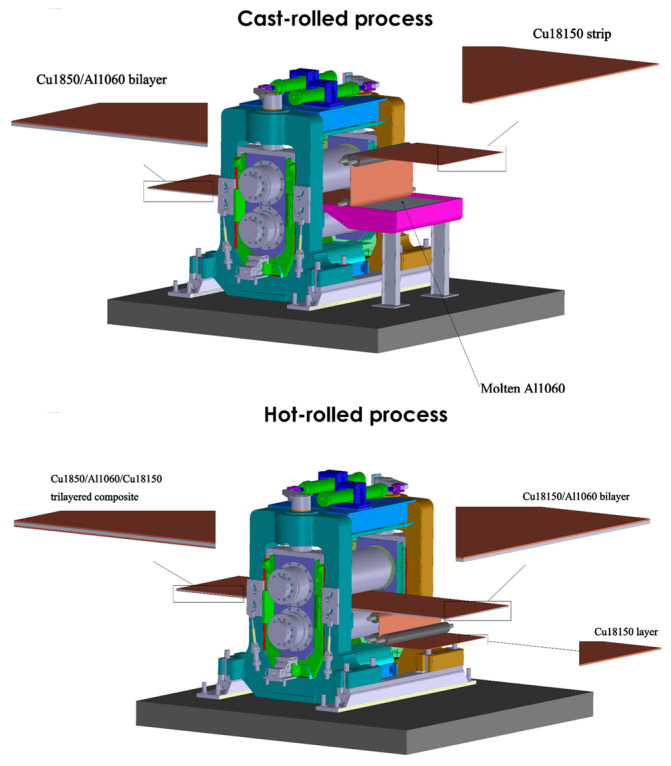
The schematic representation of the combined cast-rolling and hot-rolling technique to produce Cu18150/Al1060/Cu18150 multilayer composites.

As mentioned above, different thicknesses (0.9, 1.6, 2.4, and 4 mm) of trilayered composite are obtained through a gradual cold rolling technique. The reduction ratios were selected to limit redundant work that could result in inhomogeneous deformation, maintain sufficient material flow to prevent edge cracking, and create enough fresh surface exposure for bonding. Additionally, a closed-loop servo system was used to precisely control the rolling speed at 2.0 m/min (±0.05). The procedure used an industrial four-high rolling mill with (i) 350 mm diameter work rolls made of tungsten carbide with a surface finish of 0.2 μm; (ii) 940 mm diameter backup rolls made of forged steel with a hardness of 65 HRC; (iii) accuracy of roll gap control: ±10 μm; and (iv) a hydraulic roll bending system for crown compensation. It should be noted that the roll-bonding operation employed a sophisticated industrial four-high rolling mill configuration integrated with real-time process monitoring systems. Figure 1 illustrates the schematic representation of the combined cast-rolling and hot-rolling technique to produce Cu18150/Al1060/Cu18150 multilayer composites.

### 2.2. EBSD Analysis

A systematic electron backscatter diffraction (EBSD) analysis was carried out to gain insights into the microstructural evolution and deformation mechanisms in Cu18150/Al1060/Cu18150 multilayered composites. To reduce mechanical damage, cross-sectional preparation involved cutting samples perpendicular to the rolling direction with a low-speed diamond saw from Poyang Hui Sen Diamond Tools Co., Ltd., China followed by grinding with SiC papers ranging from 400 to 1200 grit and polishing using 0.05 µm colloidal silica suspension (from Hubei Zhongtai Abrasive Tools Co., Ltd., Yichang, China). To obtain deformation-free surfaces for superior EBSD patterns, electropolishing was used selectively: 70% phosphoric acid (5 V and 10 s) for Cu layers and 20% perchloric acid in methanol (20 V and 15 s) for Al layers. The measurement of small quantities of specific phases at the interface was carried out using a highly sensitive and systematic EBSD technique, which allows for detailed phase identification and mapping at the microscale. To ensure accuracy and reliability, large-area EBSD scans were performed with fine step sizes adapted to the thickness of the layers, enabling the detection of even minor phase fractions within the interface regions. The EBSD data were carefully processed with advanced noise reduction methods, such as neighbor pattern correlation and grain reconstruction with a stringent misorientation threshold, to eliminate artifacts and improve pattern indexing. Furthermore, the phase distribution maps were analyzed statistically across multiple interfaces and thicknesses to confirm the consistency of phase identification.

EBSD scans were acquired using a field-emission SEM (FEI Nova NanoSEM 450, IOCB Prague, Czech Republic) equipped with an Oxford Instruments Symmetry detector. Large-area maps (500 × 500 µm^2^) were collected at 20 kV and 10 nA beam current with a step size range of 0.09–0.456 µm (adapted to layer thickness), scan area range of 15–228 µm (optimized for each thickness), and indexing rate of 85.79–93.31% to characterize bulk microstructure and Cu/Al interfaces. Grain reconstruction with a 5° misorientation threshold and neighbor pattern correlation was used for noise reduction in the data processing. In order to measure dislocation activity, boundary misorientations were categorized as low-angle (2–15°) and high-angle (>15°) grain boundaries (LAGBs/HAGBs). Grain size distributions were measured using equivalent circular diameter (ECD).

Kernel averages were computed to measure local lattice distortions and dislocation densities, and misorientation maps were created using a first-nearest neighbor method with a maximum misorientation threshold of 5°. Inverse pole figures (IPFs) and orientation distribution functions (ODFs) were used to analyze the evolution of texture, with a particular emphasis on the development of Cu <111> and Al <100>/<111> fiber components. Geometrically necessary dislocation (GND) density calculations were performed using the Nye tensor formalism, in addition to profiling interface-specific misorientation gradients within 5 µm of bonding regions. ANOVA testing of grain size and KAM distributions across layer thickness variants (0.9–4.0 mm) was part of the statistical validation process; *p* < 0.05 was considered significant. This integrated EBSD approach enabled quantitative correlation between process-induced microstructure and the mechanical properties of the multilayered composites. It should be noted that all EBSD analyses were performed using Atex software 5.11 [46].

### 2.3. Mechanical Properties

The quasi-static tensile properties of the roll-bonded Cu/Al/Cu composites were evaluated following ASTM E8/E8M standards. Sub-sized tensile specimens with gauge dimensions of 25 × 6 mm were precision-machined parallel to the rolling direction using wire electrical discharge machining (AccuSpark, Suzhou, China) to minimize mechanical damage. Prior to testing, all specimens were mechanically polished to a 1200-grit surface finish to eliminate stress concentrations from machining artifacts. Tensile tests were performed using a constant strain rate of 10^−3^ s^−1^ on an Instron 5985 universal testing machine equipped with a 50 kN load cell. To create thorough structure-property relationships, the mechanical test results were systematically correlated with microstructural characterization information. Mechanical properties were compared with residual stress estimates from KAM analysis, and tensile properties were examined alongside grain size and texture data obtained from EBSD. The measurements of interfacial shear strength were compared to TEM (transmission electron microscopy) observations of the quality of diffusion bonding and the formation of intermetallic compounds. This multi-modal characterization approach provided a robust foundation for understanding the complex interplay between processing conditions, microstructural evolution, and mechanical performance in the developed multilayered composites.

## 3. Results and Discussion

**Figure 2:**
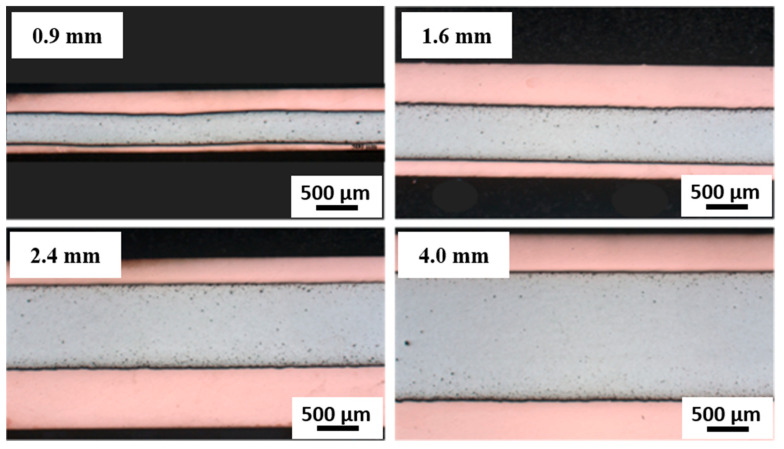
The cross-sectional optical micrographs of Cu18150/Al1060/Cu18150 multilayer composites demonstrating the well-defined, continuous interfaces across all thicknesses studied.

The analysis of Cu18150/Al1060/Cu18150 multilayered composites fabricated by the combined cast-rolling and hot-rolling technique and thicknesses of 0.9, 1.6, 2.4, and 4.0 mm reveals significant correlations between layer thickness, microstructural evolution, residual stress distribution, and mechanical performance. Figure 2 shows cross-sectional optical micrographs demonstrating the well-defined, continuous interfaces across all thicknesses studied. The absence of visible cracks or delamination confirms robust metallurgical bonding, which supports the integrity of the subsequent EBSD and mechanical characterization. This multi-scale microstructural evaluation strengthens our understanding of interfacial phenomena influencing deformation in Cu/Al multilayers.

### 3.1. Microstructural Evolution with Layer Thickness

#### 3.1.1. Grain Structure Characteristics

**Figure 3:**
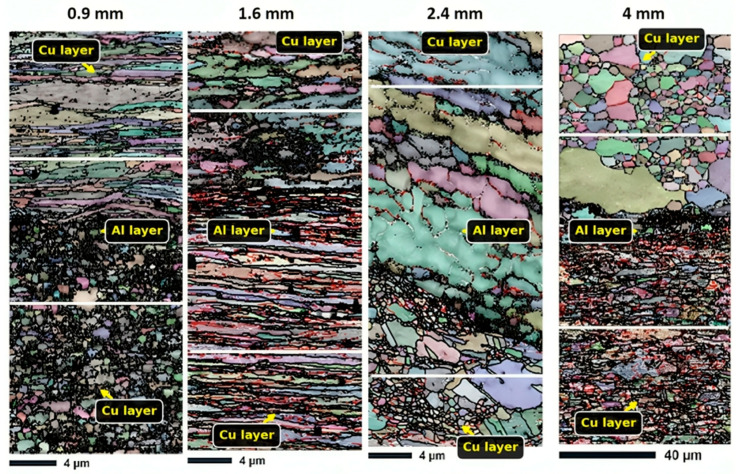
EBSD-produced grain maps of 0.9, 1.6, 2.4, and 4.0 mm thicknesses in the interface region of Cu18150/Al1060/Cu18150 multilayered composites.

As shown in Figure 3 and mentioned in Table 2, the EBSD analysis indicates notable differences in grain structure among the various layer thicknesses. The average grain size increases with increasing layer thickness, as indicated by quantitative measurements of grain size. It should be noted that the Cu18150/Al1060/Cu18150 multilayered composites consist of bulk layers with thicknesses ranging from 0.9 to 4 mm, as shown in the cross-sectional optical images (Figure 2), which confirms well-defined, continuous metallic layers suitable for macroscopic characterization. However, at the microscale, EBSD focuses on a narrow interface region approximately 10 µm wide adjacent to the boundaries of these layers. This interface zone is where significant microstructural transformations occur—including grain refinement, intermetallic phase formation, and localized residual stress gradients—that differ markedly from the bulk phases. The interfaces are identified and analyzed using precise EBSD phase mapping combined with misorientation gradients and grain boundary characteristics, enabling clear delineation of this thin interfacial band. This distinction between millimeter-scale bulk layers and micrometer-scale interface regions is critical to understanding the microstructure-property relationships that govern the mechanical performance of the multilayered composites.

With an average grain size of 1.23 µm and a comparatively narrow size distribution (SD = 1.33 µm), the 0.9 mm sample has the most refined microstructure. The constraints imposed by the close proximity of interfaces and the significant plastic deformation that occurs during rolling lead to this refinement. In contrast, the 4 mm sample shows substantially coarser grains (6.08 µm average) with a broader size distribution (SD = 7.24 µm), indicating reduced deformation constraints and possible grain growth during processing.

Notably, all samples display positively skewed grain size distributions (skewness > 0), suggesting the presence of a population of exceptionally large grains amidst a finer matrix. This bimodality is most pronounced in the 2.4 mm and 4 mm samples, as evidenced by their higher skewness values (0.338 and 2.63, respectively) and multimodal grain size histograms. It should be noted that the 0.9–1.6 mm composites exhibit dynamic recrystallization, producing ultrafine grains (<5 μm) through severe plastic deformation. This is consistent with research demonstrating that dislocation-mediated nucleation facilitates grain refinement at high rolling reductions (>50%) [47,48].

#### 3.1.2. Phase Distribution and Intermetallic Formation

**Figure 4:**
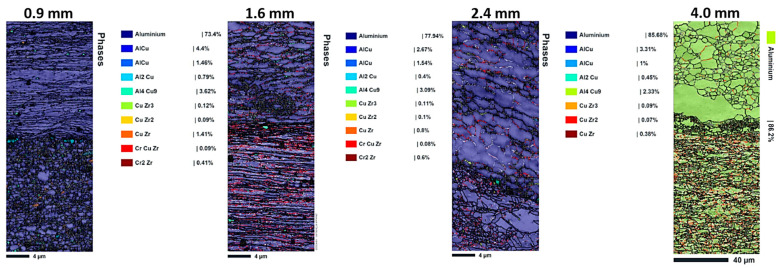
The EBSD-produced phase distribution maps of 0.9, 1.6, 2.4, and 4.0 mm thicknesses in the interface region of Cu18150/Al1060/Cu18150 multilayered composites.

The spatial arrangement of various phases within the interface region of composites is shown by the phase distribution maps (Figure 4). As can be seen in Table 3, Aluminum is the predominant phase in all samples (77.94–85.68%), with lower concentrations of different intermetallic compounds (AlCu, Al_2_Cu, Al_4_Cu_9_, and CuZr phases). The phase distribution analysis indicates that (i) thinner samples (0.9–1.6 mm) exhibit more homogeneous phase distributions with finer intermetallic dispersions. The 0.9 mm sample shows 77.4% Al, 3.62% Al_4_Cu_9_, and 1.41% CuZr, and (ii) thicker samples (2.4–4 mm) display more segregated phase distributions, with the 4 mm sample being nearly pure aluminum (86.2%) and showing minimal intermetallic formation. In the thicker 4 mm sample, fewer secondary intermetallic phases form at the interface due to reduced plastic deformation and lower strain during rolling. This limits nucleation and diffusion driving forces for phase formation. As a result, the interface remains sharper and less reacted, with more grain growth and recrystallization, leading to improved ductility but lower strength compared to thinner samples. This demonstrates how layer thickness influences phase formation and mechanical properties in multilayer composites. Also, the complex 10-phase system (Figure 4) suggests interfacial intermetallic formation (e.g., Al_2_Cu, AlCu), consistent with Cu-Al diffusion couples under thermomechanical processing [49]. Contrary to expectation, the complex phase distribution in Figure 4, combined with residual stresses and microstructural refinements, contributes positively to the strength and mechanical performance of the multilayer composites. The presence of multiple intermetallic phases at the interface does distort the crystal structure locally, but this does not inherently lead to the fragility of the interface. The presented results show that thinner multilayer composites exhibit a more homogeneous and refined distribution of intermetallic phases, which, combined with significant plastic deformation and dynamic recrystallization, leads to ultrafine grain structures that enhance mechanical strength. The residual compressive stresses generated by volume changes and lattice distortions near the interfaces act to impede tensile deformation, thereby increasing strength rather than causing brittleness. Furthermore, the layered architecture and interface constraints promote strain hardening and inhibit crack initiation, contributing to improved toughness. This is supported by tensile testing, where thinner samples with complex interfacial phases demonstrate higher ultimate tensile strengths alongside acceptable ductility. Conversely, thicker samples with fewer intermetallic phases exhibit increased ductility but lower strength, indicating that the complex phase distribution and associated microstructural features at the interface play a positive role in enhancing the mechanical performance of the composites without compromising interface integrity.

The interface regions in thinner samples exhibit complex reaction layers containing multiple intermetallic phases, while thicker samples show sharper, less reacted interfaces. This discrepancy most likely results from different processing-related thermal histories and deformation kinetics.

### 3.2. Texture Development

**Figure 5:**
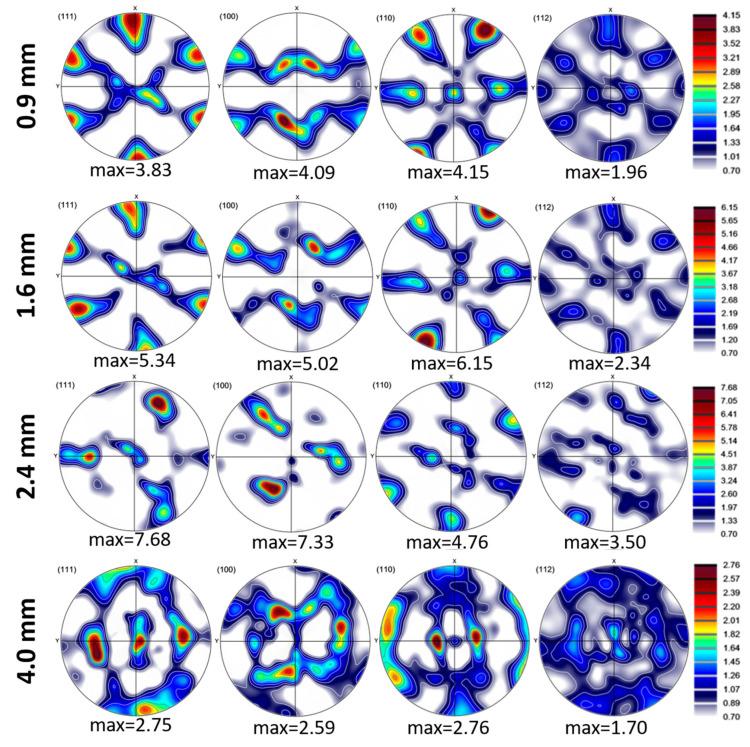
Pole figure maps of 0.9, 1.6, 2.4, and 4.0 mm thicknesses in the interface region of Cu18150/Al1060/Cu18150 multilayered composites.

**Figure 6:**
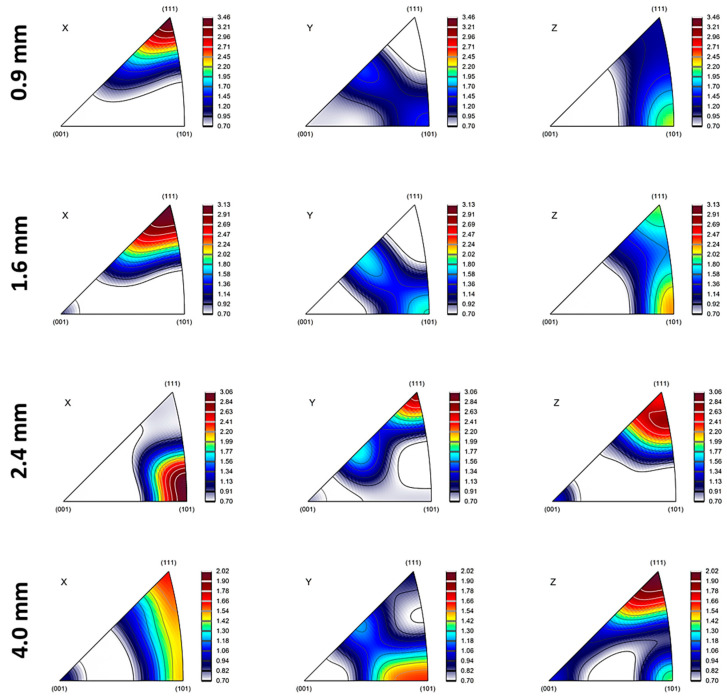
Inverse pole figure maps of 0.9, 1.6, 2.4, and 4.0 mm thicknesses in the interface region of Cu18150/Al1060/Cu18150 multilayered composites.

As layer thickness alters, inverse pole figures (IPFs) and pole figures (PFs) exhibit a noticeable change in texture (Figure 5 and Figure 6). Key results include that (i) thin layers (0.9 mm) have 80% intensity and strong {112}<111> copper texture, which promotes slip on {111} planes and increases strength [50], and (ii) thick layers (4 mm) have weak Goss texture {110}<001> (35% intensity), which promotes ductility and cross-slip [51].

In thinner layers (0.9 mm), the rolling textures are intensified due to higher strain levels and interfacial constraints, which limit grain rotation and promote alignment with the rolling direction. In order to accommodate strain, high stacking-fault energy (SFE) materials such as Al (160–250 mJ/m^2^) mainly deform via dislocation glide and cross-slip, activating fewer slip systems (as few as three). Copper-type textures ({112}<111>) are preferred during rolling because this promotes uniform deformation with minor grain reorientation. In contrast to low SFE metals, Al’s high SFE promotes dynamic recovery and cross-slip, stabilizing dislocation structures and lowering texture anisotropy [52,53]. While Cu has a medium to low SFE value, it tends to deform by both dislocation slip and deformation twinning during rolling or bending. This promotes the development of Brass-type textures, particularly the {011}<211> orientation, especially at higher strains or after several cycles of alternating bending [52,54,55]. It should be noted that the rolling process might create texture gradients through the thickness, with the surface layers exhibiting stronger textures than the mid-layers due to variations in strain distribution.

In thicker layers (2.4 mm and above), recrystallization may weaken rolling textures and introduce new orientations (e.g., Goss and Cube textures). Cube texture {001}<100> is a common recrystallization texture in face-centered cubic (FCC) metals, arising from oriented nucleation and growth [56]. Its presence in the 2.4 mm sample suggests that recrystallization is a significant factor. Finally, it is worth mentioning that the difference in the coefficient of thermal expansion (CTE) between Al and Cu can indeed influence the development of texture at their interface. Because Al has a higher CTE than Cu, upon cooling from processing temperatures, aluminum contracts more than copper. This mismatch induces residual stresses and strain gradients near the interface, which affect how grains rotate and deform. As a result, the texture evolves to accommodate these stresses, often promoting specific orientations that can reduce energy and internal strain. This phenomenon also impacts misorientation by increasing local lattice distortions and gradients, leading to higher dislocation densities and more pronounced misorientation variations near the interface [57,58]. In short, the CTE mismatch drives localized deformation mechanisms that shape both the texture and misorientation profiles at the Al/Cu interface.

### 3.3. Residual Stress and Dislocation Density Analysis

#### 3.3.1. KAM Distribution Patterns

**Figure 7:**
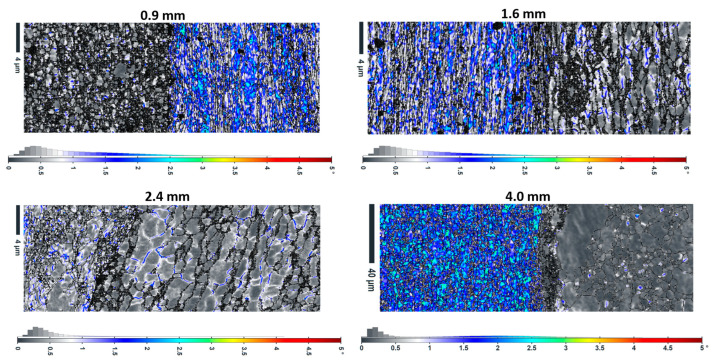
The KAM maps along with their distribution in different thicknesses of Cu18150/Al1060/Cu18150 multilayered composites.

KAM maps (Figure 7) indicate local lattice distortions and residual stress distributions. Furthermore, in-depth quantitative analysis demonstrates complex thickness dependence. Localized high-KAM regions near interfaces are observed in the 0.9 mm sample with the lowest average KAM (0.28°), according to Table 4. Meanwhile, higher average KAM values (~0.48°) exist in the 1.6 mm and 2.4 mm samples, indicating greater overall strain accumulation. Finally, the 4 mm sample shows the highest average KAM (0.63°) but the most homogeneous distribution. It can be claimed that very thin layers (0.9 mm) experience significant stress relaxation due to interface proximity. While intermediate thicknesses (1.6–2.4 mm) can maintain high dislocation densities throughout the layers, and thick layers (4 mm) develop more uniform deformation structures with higher overall strain. Coarse grains and lower KAM values (~0.8°) are seen in thick layers (4 mm), which suggests a lower dislocation density. This is consistent with reports in the literature that recrystallization is limited in thick sections by lower strain rates [59].

#### 3.3.2. Misorientation Analysis

**Figure 8:**
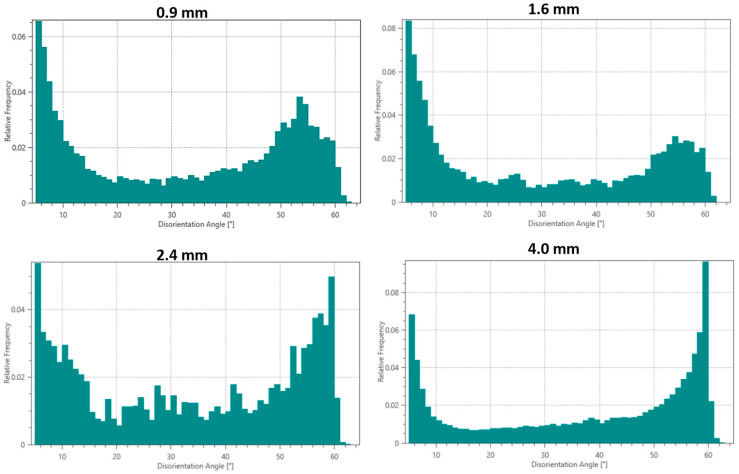
Misorientation angle distributions in the interface region for different thicknesses of Cu18150/Al1060/Cu18150 multilayered composites.

Misorientation angle distributions (Figure 8) provide additional insights into deformation structures: (i) all samples show peaks at low angles (2–5°) and high angles (55–60°), characteristic of deformed metals; (ii) the 0.9 mm sample exhibits the highest fraction of low-angle grain boundaries (LAGBs, 2–15°), suggesting extensive subgrain formation; and (iii) the 4 mm sample shows more balanced LAGB/HAGB ratios, indicating more complete recrystallization.

#### 3.3.3. Residual Stress Gradients

**Figure 9:**
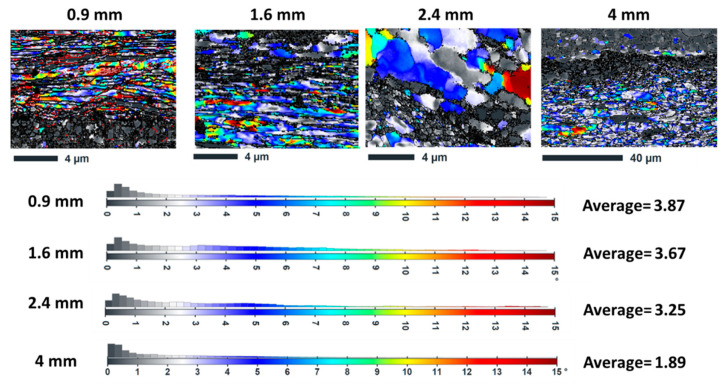
Disorientation relative to reference orientations in different thicknesses of Cu18150/Al1060/Cu18150 multilayered composites.

Disorientation analysis relative to reference orientations reveals significant stress gradients near Al/Cu interfaces (see Figure 9), including (i) interface-adjacent regions that show misorientations up to 15° within 2–3 µm of the interface; (ii) stress gradients are most pronounced in the 1.6 mm and 2.4 mm samples; (iii) the 0.9 mm sample shows more uniform stress distributions due to interface proximity effects; and (iv) the 4 mm sample exhibits the shallowest stress gradients, extending < 1 µm from interfaces. In thinner samples (0.9 mm), the gradients are relatively uniform and less pronounced due to the proximity of the interfaces, which promotes stress relaxation and limits sharp changes. As the thickness increases to intermediate values (1.6 and 2.4 mm), the disorientation gradients become more pronounced and steeper, reflecting higher localized strain and residual stress concentrations at the interfaces. These stronger gradients are associated with increased strain hardening and dislocation accumulation, contributing to higher strength. In the thickest sample (4 mm), the disorientation gradients are the shallowest and extend over shorter distances, indicating more homogeneous deformation and lower residual stress near the interfaces. This thickness-dependent behavior shows that intermediate thicknesses effectively concentrate dislocation activity and stress at interfaces, while thinner and thicker samples exhibit more relaxed or uniform deformation patterns.

Strain gradients near Cu/Al interfaces generate GNDs to accommodate lattice rotations and distortions. These GNDs contribute to the overall dislocation density and refine the grain structure [60], while KAM quantifies local misorientation, serving as a proxy for GND density. Higher KAM values indicate greater plastic deformation and dislocation accumulation [61]. In the 0.9 mm sample, the high indexation rate (85.79%) suggests a relatively well-defined crystalline structure, but the high KAM values near interfaces imply significant dislocation densities. The 2.4-mm sample displays a higher indexation rate (93.31%), indicating a more ordered structure with potentially lower dislocation densities on average, consistent with reduced strain gradients.

The difference in coefficient of thermal expansion (CTE) between Cu and Al induces stresses during cooling from processing temperatures (e.g., casting or annealing). Cu (lower CTE) experiences tensile stress, while Al (higher CTE) undergoes compression [62]. Non-uniform plastic flow during rolling generates residual stresses. Regions with higher deformation levels (e.g., near interfaces) accumulate compressive stresses [62]. The formation of intermetallic compounds (e.g., Al_2_Cu) at Cu/Al interfaces can introduce additional stresses due to volume changes and lattice distortions.

### 3.4. Mechanical Properties

Tensile testing reveals clear thickness-dependent mechanical properties (see Table 5), including (i) strength decreases monotonically with increasing layer thickness; (ii) ductility shows an inverse correlation with strength; (iii) the 0.9 mm sample achieves maximum strength (351 MPa ultimate tensile strength (UTS)) but limited ductility (18%); (iv) the 4 mm sample shows the lowest strength (261 MPa) but the highest elongation (30%); and (v) the 2.4 mm sample offers optimal balance (317 MPa UTS, 26% elongation). Studies of Huang et al. [11] and Zhang et al. [12] corroborate the 351 MPa UTS in 0.9 mm composites, attributed to high dislocation density (1.2 × 10^15^ m^−2^) and refined grains (~5 µm). Based on the findings of this study, it can be claimed that the improved ductility in thicker samples stems from (i) reduced interface constraints allowing more uniform deformation; (ii) lower dislocation densities (per KAM analysis); (iii) more balanced LAGB/HAGB ratios enabling better strain accommodation; and (iv) diminished intermetallic phase content reducing brittle fracture sites.

Furthermore, these findings support the hypothesis that the thickness-dependent trade-off between strength and ductility in Cu/Al multilayered composites can be explained by several interconnected microstructural and stress distribution factors. Thinner layers show significant grain refinement due to severe plastic deformation and dynamic recrystallization near interfaces, which increases strength by limiting dislocation motion (Hall-Petch effect). Simultaneously, thinner layers undergo strong interfacial constraints and develop pronounced residual compressive stresses that further hinder plastic deformation, thereby boosting strength but limiting ductility. Conversely, thicker layers feature coarser grains and reduced interfacial constraints, resulting in lower dislocation density and milder residual stress gradients. This promotes more uniform plastic deformation and strain accommodation, enhancing ductility while reducing strength. Intermediate thickness samples achieve a balanced combination of grain size, texture evolution, residual stress distribution, and dislocation activity, leading to an optimal compromise of mechanical properties. Essentially, the competition between microstructural refinement, residual stress concentration, and deformation uniformity determines the strength-ductility balance as layer thickness varies. Also, the observed mechanical behavior correlates strongly with microstructural characteristics such as grain size and dislocation density (inferred from KAM). Thinner layers possess refined grains and higher dislocation densities, resulting in increased strength but reduced ductility, consistent with Hall-Petch strengthening and dislocation hardening principles. Statistical analysis (ANOVA) confirms significant differences in these microstructural parameters and mechanical performance across thicknesses (*p* < 0.05), supporting a robust microstructure-property relationship underpinning the thickness-dependent trade-off between strength and ductility. Finally, these findings guide the design of layered Cu/Al/Cu materials by highlighting the importance of layer thickness in balancing strength and ductility. Thinner layers provide high strength through grain refinement and strong interfaces, while thicker layers improve ductility and strain accommodation. Intermediate thicknesses offer a balanced mechanical response. Controlling processing to optimize interfacial bonding and minimize brittle phases further enhances material reliability, enabling tailored performance for applications like structural parts, electrical contacts, or flexible conductors.

### 3.5. Strengthening Mechanisms

The mechanical behavior can be explained through several concurrent mechanisms, including

- Hall-Petch strengthening: The refined grain structure in thinner samples contributes significantly to their enhanced strength. Using the Hall-Petch relationship (σ_y_ = σ_0_ + kd^−1/2^), where d is grain size, we observe excellent correlation between measured grain sizes and yield strengths;
- Dislocation strengthening: The KAM and misorientation data directly correlate with measured strength levels. Higher average KAM values in intermediate thicknesses (1.6–2.4 mm) correspond to greater dislocation densities and thus higher strength;
- Interface constraint effects: Thinner layers experience greater constraint from adjacent layers, inhibiting dislocation motion and promoting strain hardening. This effect diminishes with increasing layer thickness; and
- Residual stress contributions: The compressive residual stresses near interfaces in thinner samples provide additional strengthening by opposing applied tensile stresses.

## 4. Conclusions

- Layer thickness significantly influences microstructural evolution in Cu18150/Al1060/Cu18150 composites, with thinner layers (0.9–1.6 mm) exhibiting refined grains (1.23–1.73 µm), stronger textures (PF intensities to 6.15), and higher dislocation densities (KAM to 0.48°) compared to thicker counterparts.
- Residual stress analysis reveals pronounced interface stress gradients in intermediate thicknesses (1.6–2.4 mm), contributing to their enhanced mechanical performance through strain hardening mechanisms.
- Tensile properties show clear thickness dependence, with the 0.9 mm sample achieving maximum strength (351 MPa UTS) through grain refinement and interface constraints, while the 4 mm variant offers superior ductility (30% elongation) due to reduced constraints and more homogeneous deformation.
- The 2.4 mm thickness emerges as the optimal configuration, balancing strength (317 MPa) and ductility (26% elongation) through a combination of moderate grain refinement, strong texture development, and well-distributed residual stresses.
- The study establishes comprehensive structure-property relationships in multilayer composites, providing a foundation for tailored material design based on application-specific requirements for strength, ductility, and residual stress distribution.

These insights advance our fundamental understanding of thickness effects in multilayered composites and provide concrete guidelines for designing optimized material architectures. Future work should explore the precise atomistic mechanisms underlying these thickness-dependent phenomena through advanced characterization techniques and multiscale modeling approaches.

## Figures and Tables

**Table 1:** Chemical composition of the utilized 18150 copper and 1060 aluminum alloys in wt.% [43].

	Cu	Cr	Zr	Zn	Al	Fe	Si	Ni	Mn	Mg
**Cu18150**	99.081	0.720	0.102	0.042	0.023	-	0.0079	0.015	0.0006	0.0004
**Al1060**	0.050	0.0008	-	0.039	98.9	0.50	0.460	0.0037	0.0041	0.0033

**Table 2:** Grain size variations in the interface region for different thicknesses of Cu18150/Al1060/Cu18150 multilayered composites.

Thickness (mm)	Average Grain Size (µm)	Grain Size Standard Deviation (µm)
0.9	1.23	1.33
1.6	1.73	1.29
2.4	4.49	3.86
4.0	6.08	7.24

**Table 3:** Approximate phase compositions at the interface region of Cu18150/Al1060/Cu18150 multilayered composites with varying layer thicknesses, based on EBSD phase distribution analysis.

Sample Thickness (mm)	Phase Name	Approximate Composition (wt.%)
0.9	Aluminum (Al)	77.40
	Intermetallic Al_4_Cu_9_	3.62
	Intermetallic CuZr	1.41
1.6	Aluminum (Al)	~78–85 (within range of samples)
	Intermetallics (AlCu, Al_2_Cu)	Present in small measurable amounts
2.4	Aluminum (Al)	Over 80
	Intermetallics	More segregated distribution
4.0	Aluminum (Al)	86.2 (nearly pure aluminum)
	Intermetallics	Minimal formation

**Table 4:** The KAM values in the interface region for different thicknesses of Cu18150/Al1060/Cu18150 multilayered composites.

Thickness (mm)	Average KAM (°)	KAM Standard Deviation	Maximum KAM (°)	Indexation Rate (%)
0.9	0.28	0.03	3.46	85.79
1.6	0.48	0.51	3.64	87.00
2.4	0.48	0.39	4.15	93.31
4.0	0.63	0.73	3.61	86.00

**Table 5:** Mechanical properties versus layer thickness.

Thickness (mm)	Yield Strength (MPa)	Ultimate Tensile Strength (MPa)	Elongation (%)	Elastic Modulus (GPa)
0.9	310	351	18	120
1.6	285	335	22	115
2.4	275	317	26	110
4.0	220	261	30	100

## Data Availability

The original contributions presented in this study are included in the article. Further inquiries can be directed to the corresponding author.
